# Optimal Binary Solvent Extraction System for Phenolic Antioxidants from Mengkudu (*Morinda citrifolia*) Fruit

**DOI:** 10.3390/molecules18067004

**Published:** 2013-06-14

**Authors:** Yin Yin Thoo, Swee Kheng Ho, Faridah Abas, Oi Ming Lai, Chun Wai Ho, Chin Ping Tan

**Affiliations:** 1 School of Hospitality, Tourism and Culinary Arts, KDU University College, Jalan SS 22/41, Damansara Jaya, 47400 Petaling Jaya, Selangor, Malaysia; E-Mail: yy.thoo@kdu.edu.my; 2 Department of Food Technology, Faculty of Food Science & Technology, Universiti Putra Malaysia, 43400 UPM Serdang, Selangor, Malaysia; E-Mail: hkheng@hotmail.com; 3 Department of Food Science, Faculty of Food Science & Technology, Universiti Putra Malaysia, 43400 UPM Serdang, Selangor, Malaysia; E-Mail: faridah@food.upm.edu.my; 4 Department of Bioprocess Technology, Faculty of Biotechnology and Biomolecular Sciences, Universiti Putra Malaysia, 43400 UPM Serdang, Selangor, Malaysia; E-Mail: omlai@biotech.upm.edu.my; 5 Department of Food Science and Nutrition, Faculty of Applied Sciences, UCSI University, No.1, Jalan Menara Gading, UCSI Heights, Cheras 56000, Kuala Lumpur, Malaysia; E-Mail: cwho@ucsiuniversity.edu.my

**Keywords:** mengkudu (*Morinda citrifolia*), total phenolic content (TPC), total flavonoid content (TFC), 2,2′-azino-bis(3-ethylbenzothiazoline-6-sulphonic acid) (ABTS) radical-scavenging capacity, 2,2′-diphenyl-1-picrylhydrazyl (DPPH) radical-scavenging capacity

## Abstract

Antioxidants have been widely used in the food industry to enhance product quality by preventing oxidation of susceptible substances. This work was carried out to maximise the recovery of total phenolic content (TPC), total flavonoid content (TFC), 2,2′-azino-bis(3-ethylbenzothiazoline-6-sulphonic acid) (ABTS) radical-scavenging capacity and 2,2′-diphenyl-1-picrylhydrazyl (DPPH) radical-scavenging capacity from *Morinda citrifolia* fruit via modification of the ethanol concentration, extraction time and extraction temperature at minimal processing cost. The optimised conditions yielded values of 881.57 ± 17.74 mg GAE/100 g DW for TPC, 552.53 ± 34.16 mg CE/100 g DW for TFC, 799.20 ± 2.97 µmol TEAC/100 g DW for ABTS and 2,317.01 ± 18.13 µmol TEAC/100 g DW for DPPH were 75% ethanol, 40 min of time and 57 °C. The four responses did not differ significantly (*p* > 0.05) from predicted values, indicating that models obtained are suitable to the optimisation of extraction conditions for phenolics from *M. citrifolia*. The relative amounts of flavonoids were 0.784 ± 0.01 mg quercetin/g of extract and 1.021 ± 0.04 mg rutin/g of extract. On the basis of the results obtained, *M. citrifolia* extract can be used as a valuable bioactive source of natural antioxidants.

## 1. Introduction

Recently, there has been a growing movement away from the use of synthetic antioxidants and toward the use of natural antioxidants. This trend is thought to be due to the adverse effects of synthetic antioxidants like butylated hydroxyanisole (BHA) and butylated hydroxytoluene (BHT) on human health [1,2]. As a result, it is interesting and meaningful to discover effective antioxidants from natural sources for food industrial, pharmaceutical and cosmetic use although they may not be comparable in efficiency to synthetic agents.

*Morinda citrifolia* (Rubiaceae), also known as noni, originated in tropical Asia or Polynesia [3]. The roots, stems, bark, leaves, flowers and fruits of *M. citrifolia* have been traditionally used as a folk remedy to treat diseases such as diabetes, hypertension and cancer [4]. With the profound nutritional and functional properties of *M. citrifolia*, its fruits are now commercialized as “noni juice”. These beneficial effects were currently believed to be linked to the fact that *M. citrifolia* is rich in phytochemicals and particularly in phenolic compounds. Recently, several novel bioactive compounds such as flavonol glycosides, iridoid glycosides and anthraquinones have been identified in the fruits of *M. citrifolia* [4,5].

Extraction is a very important stage in the recovery of bioactive compounds from natural samples, where extraction procedures must be versatile, relatively simple, inexpensive and able to both preserve and extract most of the bioactive compounds present in a plant matrix. However, there is no generalised extraction procedure that is applicable to all phenolic compounds; plant materials have diverse structures and extraction procedures can interact with other components of the plant matrix [6,7]. In addition, the extractability of phenolic compounds and their antioxidant capacities also affected by other factors including solvent composition, extraction time, extraction temperature, pH, solvent to solid ratio and the number of extraction steps [8,9,10]. Thus, extraction procedures for phenolic compounds from *M. citrifolia* must be optimised.

To the best of our knowledge and the literature search, it was revealed that only a few scientific studies has been undertaken to evaluate *M. citrifolia* to its value for the phenolics extraction. Therefore, current study was designed and undertaken to optimise extraction conditions for phenolic compounds from *M. citrifolia* to confirm and provide scientific basis on the effects of ethanol concentration, extraction time and extraction temperature as well as optimised conditions prior its incorporation into food materials.

## 2. Results and Discussion

### 2.1. Model Fitting

The responses consisting of TPC, TFC, ABTS and DPPH radical-scavenging capacities for *M. citrifolia* extract by using solvent extraction were optimised based on the central composite rotatable design (CCRD). A second-order regression equation (Equation 1) was employed to fit the experimental data, while the regression coefficients for the intercept, linear, quadratic and interaction terms of the model were statistically analysed for analysis of variance (ANOVA); the results of these analyses are shown in Table 1 and Table 2:

*k   k   k − 1 k* *Y* = *β*_0_ + ∑ *βiX_i_* + ∑ *βiiXi^2^*+ ∑ ∑*βijXiXj*
i = 1   i = 1
(1)

where *X_1_*, *X_2_*,…, *X_k_* corresponds to the independent variables affecting *Y* (namely ethanol concentration, extraction time and extraction temperature) and *β_0_*, *β_i_* (*i*= 1, 2,…, *k*;), *β_ii_* (*i*= 1, 2,…, *k*; *j*= 1,2,…, *k*) values represent the regression coefficients for intercept, linear, quadratic and interaction terms, respectively. Lastly, *k* is the number of variables. Using the determined regression coefficients, four full second-order regression equations for the concentrations of TPC (*Y_1_*), TFC (*Y_2_*), ABTS (*Y_3_*) and DPPH (*Y_4_*) were established:

*Y_1_* = 930 − 32.05*X_1_* − 8.52*X_2_* + 46.87*X_3_* − 56.47*X_1_^2^* + 0.79*X_2_^2^* − 9.89*X_3_^2^* − 11.71*X_1_X_2_* − 11.12*X_1_X_3_* + 25.55*X_2_X_3_* (2)

*Y_2_* = 513.97 + 75.63*X_1_* − 8.45*X_2_* + 43.96*X_3_* − 56.54*X_1_^2^* + 19.75*X_2_^2^* − 0.24*X_3_^2^* + 4.08*X_1_X_2_* − 21.17*X_1_X_3_* − 8.15*X_2_X_3_* (3)

*Y_3_* = 746.24 + 24.45*X_1_* + 1.33*X_2_* − 3.17*X_3_* + 14.89*X_1_^2^* + 5.92*X_2_^2^* + 14.62*X_3_^2^* + 0.31*X_1_X_2_* − 0.10*X_1_X_3_* + 2.29*X_2_X_3_* (4)

*Y_4_* = 2168.70 + 198.52*X_1_* − 5.04*X_2_* + 61.83*X_3_* + 47.96*X_1_^2^* − 57.80*X_2_^2^* − 55.31*X_3_^2^* − 8.09*X_1_X_2_* − 55.68*X_1_X_3_* − 3.67*X_2_X_3_* (5)

In Equations (2–5), *X_1_*, *X_2_* and *X_3_* correspond to the coded values of the three independent variables of ethanol concentration, extraction time and extraction temperature, respectively. Reduced second-order regression models for the four responses using significant terms for ethanol concentration (*X_1_*), extraction time (*X_2_*) and extraction temperature (*X_3_*) were as follows:

*Y_1_* = 923.18 – 32.05*X_1_* + 46.87*X_3_* – 55.64*X_1_^2^* (6)

*Y_2_* = 513.77 + 75.63*X_1_* + 43.96*X_3_* – 57.51*X_1_^2^* + 19.78*X_2_^2^* − 21.17*X_1_X_3_* (7)

*Y_3_* = 746.24 + 24.45*X_1_* + 14.89*X_1_^2^* + 5.92*X_2_^2^* + 14.62*X_3_^2^* (8)

*Y_4_* = 2168.70 + 198.52*X_1_* + 61.83*X_3_* + 47.96*X_1_^2^* − 57.80*X_2_^2^* − 55.31*X_3_^2^* − 55.68*X_1_X_3_* (9)

**Table 1:** Estimated regression coefficients of the second-order polynomial model for three dependent variables of mengkudu (*Morinda citrifolia*) crude extract.

Independent Variables	Regression coefficients				
	TPC ^a^			TFC ^b^	
	Full Quadratic Model	Reduced Quadratic Model		Full Quadratic Model	Reduced Quadratic Model
Intercept, *X*_0_	930.00	923.18		513.97	513.77
Linear					
*X*_1_, Ethanol concentration	−32.05 *	−32.05 *		75.63 *	75.63 *
*X*_2_, Extraction time	−8.52	-		−8.45	−8.45
*X*_3_, Extraction temperature	46.87*	46.87*		43.96 *	43.96 *
Quadratic					
*X*_1_^2^	−56.47 *	−55.64 *		−57.54 *	−57.51 *
*X*_2_^2^	0.79	-		19.75 *	19.78 *
*X*_3_^2^	−9.89	-		−0.24	-
Interaction					
*X*_12_	−11.71	-		4.08	-
*X*_13_	−11.12	-		−21.17	−21.17 *
*X*_23_	25.55	-		−8.15	-
Model					
F value	6.02	17.05		23.87	43.14
*p* value	0.0067	<0.0001		<0.0001	<0.0001
Lack of fit					
F value	0.99	0.93		2.27	1.61
*p* value	0.5177	0.5840		0.2239	0.3391
Mean	879.62	879.62		480.49	480.49
Standard deviation	42.79	41.82		27.89	25.35
R^2^	0.8576	0.7732		0.9598	0.9557
Adjusted R^2^	0.7151	0.7279		0.9196	0.9335
CV	4.86	4.75		5.80	5.28

^a^ Total phenolic content (TPC) (mg GAE/100g dry weight, DW). ^b^ Total flavonoid content (TFC) (mg CE/100 g dry weight, DW). * Significant at 0.05 level.

For the fitted model, the model coefficients, *F*-values and *p*-values demonstrate the significance of the experimental variables. The *p-*values were used to verify the significance of the coefficients in order to understand the mutual interactions pattern between the independent variables [11]. The high *F*-value (*F*_model_ = 6.02 − 43.14) with a low probability value for the models (*p* < 0.01) justifies the significance of each independent variables in the fitted models. Table 1 and Table 2 show that both the full and reduced second-oder regression models were highly significant (*p* < 0.01) and the lack-of-fits were insignificant (*p* > 0.05). These findings suggest that the models contain one or more important terms and do not suffer from lack-of-fits, that give excellent agreement between experimental and predicted values [12]. However, this is not conclusive evidence that the models accurately represent the data in the experimental region. Coefficients of determination (R^2^), or the ratios of the explained variation to the total variation, serve as a good measurement of the models’ overall performance. The R^2^ in the present study were observed to be 0.8596, 0.9598, 0.9583 and 0.9497 for TPC, TFC, ABTS and DPPH, respectively, in the full model; the R^2^ were 0.7732, 0.9557, 0.9554 and 0.9489, respectively, in the reduced model. In the meantime, adjusted-R^2^ should be an approximate value of R^2^ with differences that are no more than 0.1 (Table 1 and Table 2) to show the adequacy of the models obtained. However, if adj-R^2^ is significantly lower than R^2^, then one or more explanatory variables are missing from the mode1 [13]. Finally, the adequacy of the fitted response surface must be checked to ensure that the models exhibit an adequate fit and to prevent them from giving poor or misleading results. A coefficient of variation (CV) of less than 10% is desirable; this value suggests that an experimental point is reproducible with a high degree of precision that allows the recovery of a response is within the acceptable range shown in Table 1 and Table 2 [14,15]. 

**Table 2:** Estimated Regression Coefficients of the Second-order Polynomial Model for Three Dependent Variables of Mengkudu (*Morinda citrifolia*) Crude Extract.

Independent Variables	Regression coefficients				
	ABTS ^a^			DPPH ^b^	
	Full Quadratic Model	Reduced Quadratic Model		Full Quadratic Model	Reduced Quadratic Model
Intercept, *X*_0_	746.24	746.24		2168.70	2168.70
Linear					
*X*_1_, Ethanol concentration	24.45 *	24.45 *		198.52 *	198.52 *
*X*_2_, Extraction time	1.33	1.33		−5.04	-5.04
*X*_3_, Extraction temperature	−3.17	−3.17		61.83 *	61.83 *
Quadratic					
*X*_1_^2^	14.89 *	14.89 *		47.96 *	47.96 *
*X*_2_^2^	5.92 *	5.92 *		−57.80 *	−57.80 *
*X*_3_^2^	14.62 *	14.62 *		−55.31 *	−55.31 *
Interaction					
*X*_12_	0.31	-		−8.09	-
*X*_13_	−0.10	-		−55.68 *	−55.68 *
*X*_23_	2.29	-		−3.67	-
Model					
F value	22.97	42.84		18.90	29.21
*p* value	<0.0001	<0.0001		<0.0001	<0.0001
Lack of fit					
F value	0.80	0.57		3.12	2.27
*p* value	0.6031	0.7703		0.1466	0.2233
Mean	771.94	771.94		2119.01	2119.01
Standard deviation	8.29	7.43		66.39	60.53
R^2^	0.9583	0.9554		0.9497	0.9489
Adjusted R^2^	0.9166	0.9331		0.8995	0.9165
CV	1.07	0.96		3.13	2.86

^a^ ABTS radical scavenging capacity (µmol TEAC/ 100 g dry weight, DW). ^b^ DPPH radical scavenging capacity (µmol TEAC/100 g dry weight, DW). * Significant at 0.05 level.

The R^2^ values for TFC, ABTS and DPPH in both the full and reduced regression quadratic models were at least 90%; this suggests that a high proportion of variability can be attributed to the three independent variables and that a very small proportion of variability in the response variables is due to other uncontrollable factors. However, the R^2^ values for both the full and reduced regression quadratic models of TPC were below 90%. This suggests that the recovery of TPC may be affected by other factors beyond the three independent variables investigated in this study. This finding may be due to problems with the TPC assay; the assay may overestimate TPC by reacting with interfering substances and non-phenolic compounds that are present in crude extracts [16]. Consequently, further study of the purification of crude extracts is needed so that impurities can be removed from the crude extract after the optimisation of the solvent extraction system. Although R^2^ for the TPC model was less than 90%, it was reasonably close to 0.80 (80%) and met the other criteria mentioned [17,18]. Therefore, the models for TPC, TFC, ABTS and DPPH were judged to accurately represent the data in the experimental region and were used to navigate the design space.

Overall, the regression coefficients for all four responses are similar in both the full and reduced regression quadratic models. We therefore believe that the removal of non-significant terms from the full regression quadratic model using a backward elimination process strongly influenced the significance of the experimental variables and, to a lesser extent, also influenced the regression coefficients. Therefore, reduced regression quadratic models with enhanced experimental variables were used to interpret the experimental data. 

The correlations between the level of phenolic compounds and their antioxidant capacities with regard to the experimental runs were tabulated in Table 3. Positive significant (*p* < 0.05) were found between DPPH with TFC and DPPH with ABTS. In contrast to the findings reported by Thoo *et al*. [19] on the strong significant positive correlation between TPC and DPPH, current findings showed an insignificant negative correlation. This finding is attributed to the use of CCRD for the optimisation process that refer the correlation between the responses studied at all level of experimental runs instead of single factor experiment that enable correlation between responses under the influence of one extraction parameter at a time. Thus, this study demonstrated that the optimisation method overcomes the limitation of classical single factor experiments and provide statistically reliable results for the production of extract at optimised process parameters.

**Table 3:** Correlation between assays under influence of different experimental runs (*n* = 2).

r	TPC	TFC	ABTS
TFC	0.396		
ABTS	−0.637*	0.147	
DPPH	−0.228	0.522 *	0.488 *

### 2.2. Effects of Ethanol Concentration, Extraction time and Extraction Temperature

#### 2.2.1. Phenolic Compounds (TPC and TFC)

Phenolics are widely distributed and found in large quantity in the plant kingdom. According to recent reports, phenolic compounds may contribute to the overall antioxidative action [19,20]. As efficient radical chain-breaking agents, phenolics play an important role in stabilising lipid peroxidation by delocalizing the unpaired electron [20]. Therefore, TPC and TFC assays were determined in this study as they are important in various food materials. 

**Figure 1:**
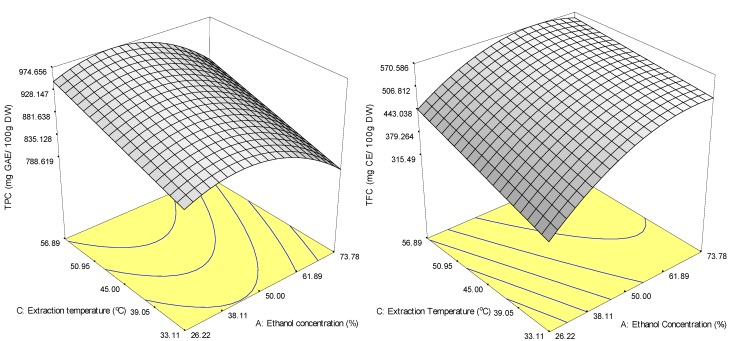
Response surface plot for the effects of extraction conditions on the recovery of TPC (**a**) and TFC (**b**) of ethanol extracts from *M. citrifolia* powder by solvent extraction.

ANOVA revealed that interaction between the solvent and extraction time did not have a significant effect (*p* > 0.05) on either TPC or TFC recoveries. The response surface was therefore generated as a 3-dimensional plot of ethanol concentration and extraction temperature while extraction time was held constant at 70 min (Figure 1a). The reduced model (Equation 6) indicated that the extraction temperature had the most linear effect on the recovery of TPC as it showed the largest positive linear coefficient (46.87). In contrast, ethanol concentration showed the largest negative linear coefficient (‒32.05). The linear and quadratic effects on TPC that explain the nature of the curve are shown in Figure 1. The response surface plots (Figure 1a) show that TPC increased slowly with the increase in ethanol concentration at a fixed extraction time (70 min) and reached a peak with 43.5% ethanol at 49.2 °C. However, further increases in the ethanol concentration caused the trend to reverse, even at higher extraction temperatures. These findings reveal that most of the phenolics in *M. citrifolia* extract is polar in nature and is attributed to the chemical structure of phenolic compounds which contain one or more hydrophilic hydroxyl groups [21].

Based on Equation (7), the response surface plot represents the effects of the independent variables on TFC from *M. citrifolia* (Figure 1b). Ethanol concentration had a highly significant (*p* < 0.001) influence on the response surface and had the highest positive regression coefficient (75.63); extraction temperature had the next highest positive regression coefficient (43.96) and was followed by quadratic extraction time (19.78).

The plot of TFC as affected by ethanol concentration and extraction temperature (Figure 1b) shows a linear increase in TFC with an increase in ethanol concentration at a fixed temperature until a stationary point is reached at 67.8% ethanol; TFC also increased significantly (*p* < 0.05) with the increase in extraction temperature at a fixed ethanol concentration.

The positive parabolic shape of the curve when ethanol concentration was increased at a fixed extraction time and temperature reconfirms the negative quadratic effect of ethanol concentration. Other studies have demonstrated that ethanol concentration is important to the extraction of phenolic compounds from various plant products [22,23,24], while extraction temperature can enhance the recovery of phenolic compounds [22,23,24]. In other words, both ethanol concentration and extraction temperature played important roles in the extraction of phenolic compounds (TPC) from *M. citrifolia*. 

Extraction temperature affected the extraction of TFC and TPC similarly, but ethanol concentration did not. This suggests that the TFC and TPC from *M. citrifolia* are of different compounds where the variation in the yields is likely attributed to the polarities of the bioactive compounds [25]. Zhang *et al*. found that only a solvent with a polarity that is compatible with a particular bioactive compound can extract it from the plant matrix. This phenomenon had complicated the process to develop a standard extraction solvent suitable for the extraction of all plant bioactive compounds [26]. A positive quadratic effect of extraction time was obtained for TFC as well. The flavonoids thus seem to need a longer extraction period to be delivered by the plant materials. Most of the interactions between the variables had a very minor influence on the recovery of TFC (*p*-values were larger than 0.05); the exception was *X_1_X_3_* (the interaction parameter between ethanol concentration and extraction temperature), which had a negative effect (−21.27) on the recovery of TFC. This implies that to increase the recovery of TFC, the level of one factor must increase, while the other must decrease. Figure 1b shows that TFC yield increased as ethanol concentration increased at a low extraction temperature. From the industrial point of view, a higher ethanol concentration at a lower extraction temperature is preferable since higher extraction temperatures increase costs and can make an extraction procedure uneconomical. 

#### 2.2.2. Antioxidant Capacity (ABTS and DPPH Radical-Scavenging Capacities)

The scavenging ability of phenols due to their hydroxyl group that possesses antioxidant capacities were determined by ABTS and DPPH radical-scavenging capacity in vitro due to easy operation and repeatable [27]. The positive quadratic effects of the independent variables indicate that the ABTS values decrease with increase in the independent variables (ethanol concentration, extraction time and extraction temperature) until a minimum point is reached. The ABTS values then increase with a further increase in the independent variables up to 90%, 120 min and 65 °C, respectively. This finding suggests that bioactive compounds that exhibit ABTS radical-scavenging capacity require a non-polar solvent, a more energetic condition and a longer extraction time to be extracted from the plant matrix. Elevated temperature provides energetic conditions that promote rapid mass transfer with the decreased liquid viscosity and density [28]. In other words, the rate of extraction of the chemically stable bioactive compounds from *M. citrifolia* which are responsible for the ABTS radical-scavenging capacity at higher ethanol concentration and elevated temperature for a longer period is higher than the rate of decomposition of less stable bioactive compounds [8].

**Figure 2:**
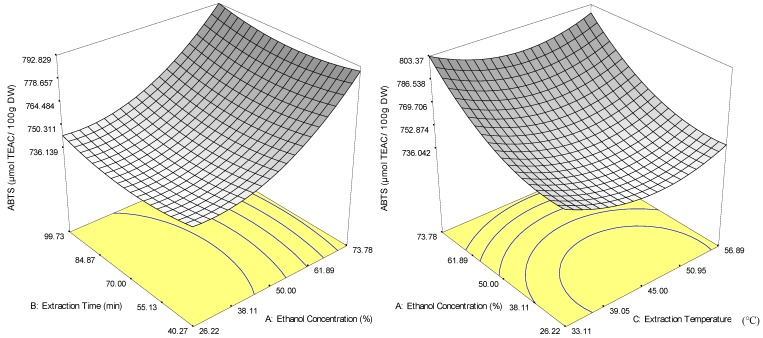
Response surface plot for the effects of extraction conditions on the recovery of ABTS of ethanol extracts from *M. citrifolia* powder by solvent extraction.

Among the significant terms, ethanol concentration had the highest regression coefficient (24.45, *p* < 0.05) and the strongest effect on the extraction of bioactive compounds that are responsible for the radical-scavenging capacity of ABTS. Figure 2a shows that ABTS values increased significantly (*p* < 0.05) when ethanol concentration was increased at a fixed extraction time but only increased slightly when extraction time was increased at a fixed ethanol concentration. Similarly, ABTS values also increased with an increase in ethanol concentration at a fixed temperature and a minor increase in extraction temperature at a fixed ethanol concentration (Figure 2b). At the same time, the quadratic terms of the three independent variables showed a positive effect on the recovery of ABTS; the highest values were observed in ethanol concentration (*X_1_^2^*, 14.89), followed by extraction temperature (*X_2_^2^*, 14.62) and extraction time (*X_3_^2^*, 5.92).

**Figure 3:**
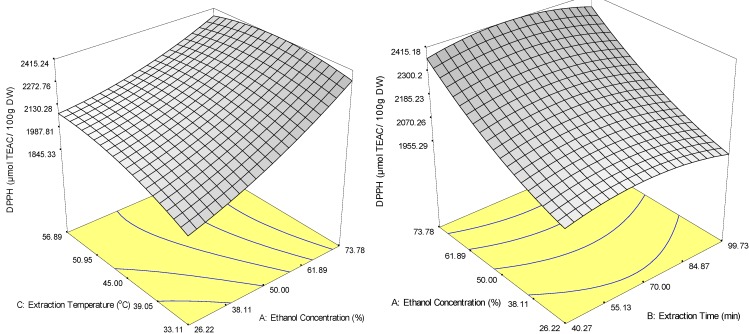
Response surface plot for the effects of extraction conditions on the recovery of DPPH of ethanol extracts from *M. citrifolia* powder by solvent extraction.

In the case of DPPH, ethanol concentration had the strongest positive impact with the highest regression coefficient (198.52); extraction temperature had the next strongest positive impact and regression coefficient (61.83) (Table 2). This shows that DPPH increased with either an increase in ethanol concentration or extraction temperature (Figure 3a), but that it was less affected by extraction time (Figure 3b). The quadratic effect of the independent variables was also observed in DPPH, but the quadratic effect of extraction time and temperature was different than with ABTS. Extraction time had the strongest negative effect (−57.80) followed by extraction temperature (−55.31). However, ethanol concentration had a positive quadratic effect (47.96). The negative quadratic effects of extraction time and temperature indicate that DPPH recovery was maximised at about 48.3 °C (Figure 3a) and 70 min (Figure 3b).

Similar to the response of TFC, only interaction between ethanol concentration and extraction temperature (*X_1_X_3_*) reached significance (*p* < 0.05) for DPPH (−55.68). According to these experimental data, TFC may contribute to the antioxidant capacity exhibited by DPPH since both showed similar trends in relation to the extraction variables. Figure 3a shows that the recovery of DPPH increased at increased ethanol concentrations and low extraction temperatures. This is consistent with previous findings that thermolabile antioxidants such as phenolic glycosides and flavonols from strawberry fruits or bioactive compounds that were mobilized at lower temperatures were degraded at higher temperature.

Overall, antioxidant capacity (ABTS and DPPH) is more sensitive and involved linear, quadratic and interaction terms of the independent variables since the accumulation of various compounds could exhibit antioxidant capacity [29]. An analysis of response surface plots of all the responses demonstrates that ethanol concentration had the most significant influence on the extraction of phenolic compounds and antioxidant capacity from *M. citrifolia* fruits; extraction temperature had the second most significant influence and extraction time had the third most significant influence. These findings are probably explained by the mechanism by which ethanol concentration directly influenced the internal parameters of the extraction system by affecting the type of bioactive compounds that were extracted [30]. Meanwhile, extraction temperature and extraction time manipulate external parameters (like the diffusion coefficient and solubility) to contribute to response recovery [31,32].

### 2.3. Optimisation and Model Verification

The optimal values of the independent variables were obtained by solving second-order regression equations (Equations 6–9) using a numerical optimisation method. Experimental data suggested the existence of optimal conditions for the solvent extraction of phenolic antioxidants from *M. citrifolia*. The optimisation of both phenolic compounds and antioxidant capacities occurred with 74.59% ethanol for 40.27 min at 56.87 °C (Table 4).

The models’ predictive values were evaluated by using *T*-test. The *T*-test was conducted to compare the experimental values with predicted values for each response. Since no significant (*p* > 0.05) difference was observed between those values, thus it verified that the response equations are adequate for predicting the yield of phenolic compounds and antioxidant capacity as a function of extraction condition for mengkudu fruit. In comparison with recent study by Thoo *et al*. [19] on investigation into the effect of extraction parameters on phenolic antioxidants from mengkudu by using single factor experiments, the yields of TFC and antioxidant capacity recoveries were improved by using RSM. The improved yield and recoveries are likely attributed to the use of higher ethanol concentration at lower temperature for longer time, whereby the optimum extraction by using single factor experiment were 40% ethanol for 80 min at 65 °C.

**Table 4:** Optimum conditions, predicted and experimental values of responses on extraction of *Morinda citrifolia* crude extract *^a^*.

Independent variables			Dependent variables (Responses)	Optimum value	
*X*_1_	*X*_2_	*X*_3_		Experimental *^b^*	Predicted
74.59	40.27	56.87	*Y*_1_	881.57 ± 17.74	883.55
			*Y*_2_	552.53 ± 34.16	583.41
			*Y*_3_	799.20 ± 2.97	801.12
			*Y*_4_	2317.01 ± 18.13	2311.27

*^a^* *X*_1_, ethanol concentration (%, v/v); *X*_2_, extraction time (min); *X*_3_, extraction temperature (^o^C); *Y*_1_, TPC (mg GAE/100 g DW); *Y*_2_, TFC (mg CE/100 g DW); *Y*_3_, ABTS radical-scavenging capacity (µmol TEAC /100 g DW); *Y*_4_, DPPH radical-scavenging capacity (µmol TEAC /100 g DW). *^b^* Mean ± standard deviation (SD) of six determinations (n = 6) from two crude extract replications.

### 2.4. Quantitative Analysis of Quercetin and Rutin in Optimised extract.

The identification of quercetin and rutin were based on retention time, UV spectra of the standards under the same conditions using HPLC-DAD. The results obtained demonstrated that the flavonoids content in the optimised solvent-extracted *M. citrifolia* extract were 0.784 ± 0.01 mg quercetin/g of extract and 1.021 ± 0.04 mg rutin/g of extract. The calibration curves were obtained by the external standard method on six level of concentration of standard, with two injections per level. Chromatogram peak areas on 360 nm for quercetin and rutin were plotted against the known concentration of the standard solutions to establish the calibration equations. The linear regression analytical data for calibration plots showed a good linear relationship with *r^2^* > 0.98 within the test ranges. The relationship between peak areas (*y*) and concentrations (*x*) was *y* = 227.22*x* − 113.6 (quercetin), *y* = 73.29*x* + 0.0039 (rutin). 

## 3. Experimental

### 3.1. Chemicals

The compounds 2,2′-azino-bis(3-ethylbenzothiazoline-6-sulphonic acid) diammonium salt (ABTS, ≈98% purity), 2,2′-diphenyl-1-picrylhydrazyl (DPPH, 95% purity), potassium persulphate (≥98% purity), sodium nitrite and (+)-catechin hydrate (≥98% purity) were purchased from Sigma-Aldrich (Steinheim, Germany). Folin-Ciocalteu’s phenol reagent, sodium carbonate (≥99.9% purity) and a sodium hydroxide solution (1 mol/L, 1 N) were purchased from Merck (Darmstadt, Germany). Gallic acid (98% purity) and Trolox (97% purity) were purchased from Acros Organics (Morris Plains, NJ, USA). Absolute ethanol (≥99.4%, v/v), denatured absolute ethanol and aluminum chloride-6-hydrate (>99 purity) were purchased from Fisher Scientific Co. (Leicestershire, UK). All of the reagents were analytical grade and all stock solutions were prepared with purified deionised water (MilliQ purification system, Millipore, Bedford, MA, USA).

### 3.2. Plant Materials

Powdered dried fruits of *M. citrifolia* were purchased from a local producer, Ethno Resources Sdn. Bhd. (Selangor, Malaysia). The *M. citrifolia* powder was vacuum packed in nylon-linear low-density polyethylene pouches and stored in the dark at ambient temperature (25 ± 3 °C) until used for further analysis.

### 3.3. Extraction of Plant Material

Dried fruits of *M. citrifolia* were extracted at solid-to-solvent ratio of 1:10 in a temperature-controlled water bath shaker (Model WNB 7-45, Memmert, Schwabach, Germany) at a constant speed; times and temperatures varied according to the study conditions. The crude extracts were cooled to room temperature with running water and filtered through Whatman No. 1 filter paper. The crude extracts were collected in amber bottle before the total phenolic content (TPC), total flavonoid content (TFC), ABTS radical-scavenging capacity and DPPH radical-scavenging capacity were determined. Each extraction was replicated; all measurements were assayed in triplicate and the data were reported as mean ± SD. Before the RSM was employed, preliminary extraction trials using a one-factor-at-a-time approach were carried out to determine the approximate optimal conditions for the extraction variables (*i.e.*, ethanol concentration, extraction time and extraction temperature). These trials used ethanol in the range of 0 to 100% (v/v) for 20 to 120 min at temperatures of 25 to 65 °C.

### 3.4. Experimental Design

#### 3.4.1. Response Surface Procedures

Response surface methodology (RSM) was used to optimise the solvent extraction of phenolic compounds from *M. citrifolia*. A CCRD was used to investigate the effects of three independent variables (the extraction variables) on the response variables (*Y_n_*) of the crude extracts (Table 5). The three independent variables were ethanol concentration (*X_1_*, 10–90% v/v), extraction time (*X_2_*, 20–120 min) and extraction temperature (*X_3_*, 25–65 °C). The independent variables were each coded at five levels (−α, −1, 0, +1, +α) and their values were selected on the basis of preliminary experiments that examined TPC (*Y_1_*), TFC (*Y_2_*), ABTS (*Y_3_*) and DPPH (*Y_4_*). The complete study design consisted of 20 experimental runs with eight factorial points, six axial points and six replications at the centre points. 

**Table 5:** Levels of independent variables established according to the central composite rotatable design (CCRD).

Independent variables	Units	Coded levels				
		−α	−1	0	+1	+α
		Actual levels				
Ethanol concentration, *X_1_*	% v/v	10	26.22	50	73.78	90
Extraction time, *X_2_*	min	20	40.27	70	99.73	120
Extraction temperature, *X_3_*	°C	25	33.11	45	56.89	65

#### 3.4.2. Determination of the Optimum Conditions and Validation of the Model

The optimal independent variables (extraction conditions) for the maximised responses of *Y_1_*, *Y_2_*, *Y_3_* and *Y_4_* were obtained using desirability function approach on the second-order polynomial model of RSM. In Design-Expert software package, each response *Y_n_* was first converted into individual desirability function *d_n_*_,_ where 0 ≤ *d_n_* ≥ 1 [33]. The value of *d_n_* increase as the desirability of the corresponding response increases. This individual desirability was combined using the geometric mean. According to Myers and Montgomery [33], if the target, T for the response *Y* is a maximum value Equation (10) was used.


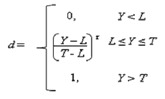
(10)

When the weight r = 1, the desirability function is linear. Selecting r > 1 places more emphasis on being close to the target value and selecting 0 < r < 1 makes this less important. Verification experiments were carried out to confirm and validate the fitted second-order polynomial regression models obtained. 

### 3.5. Analysis of the Response Variables

#### 3.5.1. Total Phenolic Content (TPC)

The TPC of the extracts was determined according to the protocol described by Li *et al*. [34] and Yoo *et al*. [35] with minor modifications. Crude extract were diluted 20 times with deionised water and a 1 mL aliquot of each diluted crude extract was mixed with 1 mL of Folin-Ciocalteu reagent (diluted 10-fold) in 15-mL aluminum foil-wrapped test tubes for 10 s using a vortex mixer (VTX-3000L, LMS, Tokyo, Japan). After 3 min, 800 µL of sodium carbonate (7.5%, w/v) was added to each test tube, and the test tubes were then vortexed for 10 s. After 2 h incubation in the dark at room temperature, the absorbance at 765 nm was determined using a Uvi light spectrophotometer (Model XTD 5, Secomam, Domont, France). A standard curve with serial gallic acid solutions (10–70 mg/L) was used for calibration. Analyses of the crude extracts were completed in triplicate and TPC was expressed as milligram of gallic acid equivalents (GAE) per 100 gram of dry weight (DW).

#### 3.5.2. Total Flavonoid Content (TFC)

The TFC of the crude extract was estimated according to the procedures described by Liu *et al*. [36] and Arancibia-Avila *et al*. [37] with slight modifications. Diluted crude extract (approximately 0.25 mL, diluted 20-fold) was mixed with deionised water (1.25 mL) and 5% sodium nitrite (75 µL) in a 15-mL aluminum foil-wrapped test tube. After 6 min, 10% aluminum chloride hexahydrate (150 µL) were added to the mixture. In the next 5 min, a 1 M sodium hydroxide solution (0.5 mL) and deionised water (275 µL) were added to the mixture. A blank was prepared by replacing crude extract (0.25 mL) with deionised water (0.25 mL). The test tubes were well mixed using a vortex mixer (VTX-3000L, LMS) for 10 s and the absorbance was measured immediately at 510 nm using Uvi light spectrophotometer (Model XTD 5, Secomam). Each crude extract was analysed in triplicate and TFC was expressed as milligram of catechin equivalent per 100 gram dry weight (mg CE/100g DW). The calibration curve was prepared with 50–800 µg/mL of catechin (*y* = 0.0033*x*; R^2^ = 0.9991).

#### 3.5.3. ABTS Radical-Scavenging Capacity

The spectrophotometric analysis of ABTS radical-scavenging capacity was carried out according to the procedures outlined by Cai *et al*. [38], Surveswaran *et al*. [39] and Saafi *et al*. [40] with slight modifications. The ABTS radical solution was produced by gently mixing a 7 mM ABTS solution (10 mL) with a 2.45 mM potassium persulphate solution (10 mL) in an amber bottle. This mixture was then stored in the dark at room temperature for 12–16 h and produced a dark blue solution. This dark blue ABTS radical solution was adjusted with denatured absolute ethanol to an absorbance of 0.7 (±0.02) at 734 nm using a Uvi light spectrophotometer (Model XTD 5, Secomam). The crude extract (approximately 100 µL) was added to the adjusted ABTS radical solution (3.9 mL) in 15-mL aluminum foil-wrapped test tubes. The test tubes were then mixed thoroughly with a vortex mixer (VTX-300L, LMS) for 10 s and allowed to stand at room temperature for 6 min. A decrease in absorbance at 734 nm was measured for each crude extract relative to the absorbance of a blank (ethanol). The absorbance of a negative control (100 µL of denatured absolute ethanol and 3.9 mL of ABTS radical solution) was also measured at 734 nm. Measurements of both the samples and the negative control were carried out in triplicate. The percentage of ABTS radical-scavenging capacity was calculated using Equation 11. ABTS was expressed as micromoles of Trolox equivalents antioxidant capacity (TEAC) using the equation obtained from the standard curve of prepared Trolox (0.1–0.8 mM) with *y* = 120.1142*x* (R^2^ = 0.9984):

ABTS radical-scavenging capacity (%) = [1 − (*A_e_* − *A_c_*)] × 100% (11)

where *A_e_* is the *A_734_* in the presence of crude extract in the ABTS radical solution and *A_c_* is the *A_734_* of the control solution (containing denatured absolute ethanol and ABTS radical solution). 

#### 3.5.4. DPPH Radical-Scavenging Capacity

The DPPH radical-scavenging capacity of crude extracts were determined according to the procedures outlined by Cai *et al*. [41], Pérez-Jiménez and Saura-Calixto [42] and Faller and Fialho [43] with slight modifications. Briefly, crude extract (100 µL) was added to absolute ethanolic DPPH (3.9 mL, 60 µM). Soon after vortexing the reaction mixture for 1 min, the test tubes were placed in dark at room temperature for 30 min before absorbance was measured at 517 nm using a Uvi light Spectrophotometer (Model XTD 5; Secomam). Simultaneously, the absorbance of the negative control (100 µL of absolute ethanol and 3.9 mL of ethanolic DPPH) was also measured at 517 nm. Each sample and negative control was assayed in triplicate. The DPPH radical-scavenging ability of the crude extract was calculated using Equation (12). Measurements were calibrated according to a standard curve of prepared Trolox (0–2.5 mM) with *y* = 37.284*x* (R^2^ = 0.9997); antioxidant capacity was expressed as micromoles of TEAC per 100 gram DW:

DPPH radical-scavenging capacity (%) = [1 − (*A*_e_ − *A*_c_)] × 100% (12)

where *A_e_* is the *A_517_* in the presence of crude extract in an absolute ethanolic DPPH solution and *A_c_* is the *A_517_* of the control solution (containing absolute ethanol and absolute ethanolic.

#### 3.5.5. Instrumentation

HPLC-DAD quantitative analysis was performed using Agilent Infinity 1200 series HPLC system (Agilent Technologies, Palo Alto, CA, USA) equipped with vacuum degasser, a quaternary pump, autosampler and ultraviolet-visible (UV-vis) multi-wavelength detector, controlled by Agilent Chemstation software. The separation was achieved on a Hypersil Gold C_18_ column (3 μm, 150 mm × 2.1 mm) at 40 °C [44,45]. The flow rate was set at 200 μL/min and sample injection volume was 10μL. The analytes were separated at gradient elution system consisted (A) deionised water containing 0.1% formic acid and (B) acetonitrile containing 0.1% formic acid as mobile phases. The gradient was as follows: 0–5 min, 95% A; 5–10 min, 90% A; 10–15 min, 80% A; 15–35 min, 55% A; 35–40 min, 95%, and finally reconditioning the column with 95% A isocratic for 5 min. Extract was prepared at 1 mg/mL and reference compounds were at appropriate concentrations for the construction of calibration curve. For each compound, at least six concentrations of the solution were analysed in duplicates. The monitoring wavelength was at 360 nm for quercetin and rutin with the scan range from 200 to 600 nm. Quantification determination was carried out using calibration curves of standards or reference standards in combinations of retention time and spectral matching. Standard calibration curves were prepared at six different concentrations (0.1–10 mg/L). Analysis was performed in duplicate for each sample and area linear regression was generated. The amount of the compound was finally expressed in mg/g of extract.

### 3.6. Statistical Analysis

Results were expressed as the mean ± standard deviation of the replicate solvent extractions and triplicate of assays. Statistical analysis was performed using Design Expert (release 6.0.10; State-Ease, Inc., Minneapolis, MN, USA). An analysis of variance (ANOVA) was conducted to determine the significance levels defined at p < 0.05, p < 0.01 and p < 0.001. Pearson correlations between variables were established using Minitab (release 14.1; Minitab Inc., State College, PA, USA).

Replicate extractions were performed at all points in the study design. The corresponding crude extracts were analysed for the dependent variables (responses) TPC (*Y_1_*), TFC (*Y_2_*), ABTS radical-scavenging capacity (*Y_3_*) and DPPH radical-scavenging capacity (*Y_4_*). Mean values were analysed using least-square regression to fit the following generalised second-order polynomial model (Equation 1) to all of the dependent Y variables [28]. 

Response surfaces plots were developed using reduced fitted polynomial models. This allows the relationship between the response and experimental levels of each factor to be examined and the optimum conditions to be identified. 

## 4. Conclusions

This study emphasised the importance of extraction conditions on the yield of phenolic compounds and antioxidant capacity recovery. It has been noticed that phenolic compounds are preferably extracted at lower ethanol concentration with higher extraction temperature while antioxidant capacities were maximised at high ethanol concentration for lower extraction temperature. The optimisation on the solvent extraction of phenolic antioxidants from *M. citrifolia* is crucial before further study on their incorporation into foods as functional ingredients. Each response demonstrated different correlation with different extraction conditions hence, selection of the optimised extraction conditions were dependent on the characteristics of bioactive compounds present as well as goals of the optimisation. The optimised conditions for the optimal phenolic compounds yields and antioxidant capacities recoveries were by using 75% ethanol and subject to extraction for 40 min at 57 °C. . Since there is no significant (*p* > 0.05) difference between experimental and predicted results, thus these verified that the final reduced response models were adequate to reflect the expected optimisation, indicating its reliability to optimise the extraction of bioactive phenolics from *M. citrifolia*. 
